# Effect of biochar on antibiotics and antibiotic resistance genes variations during co-composting of pig manure and corn straw

**DOI:** 10.3389/fbioe.2022.960476

**Published:** 2022-07-22

**Authors:** Zhenye Tong, Fenwu Liu, Yu Tian, Jingzhi Zhang, Hui Liu, Jiaze Duan, Wenlong Bi, Junmei Qin, Shaozu Xu

**Affiliations:** ^1^ College of Resources and Environment, Shanxi Agricultural University, Jinzhong, China; ^2^ Nongshengyuan Family Farm, Jinzhong, China

**Keywords:** biochar, antibiotics, antibiotic resistance genes, mobile genetic elements, compost

## Abstract

Pig manure is a reservoir of antibiotics and antibiotic resistance genes (ARGs). The effect of biochar on the variations in physicochemical properties, bacterial communities, antibiotics, ARGs, and mobile genetic elements (MGEs) of compost product during co-composting of pig manure and corn straw have been investigated in this study. Compared with the control treatment (CK), biochar addition accelerated the increase in pile temperature and prolonged the high temperature period (>55°C) for 2 days. Under biochar influence, organic matter degradation, NH_4_^+^-N conversion and NO_3_^−^-N production was accelerated, and dissolved total organic carbon (DOC) and dissolved total nitrogen (DTN) utilization by microorganisms were enhanced. Biochar addition altered the microbial community and promoted the vital activity of *Actinobacteria* in the later composting stage. The antibiotics removal efficiency (except danofloxacin and enrofloxacin) was accelerated in the early composting stage (1–14 days) by biochar addition, the pile temperature had a positive effect on antibiotics removal, and the total antibiotics removal efficiency in CK and CK+Biochar treatments was 69.58% and 78.67% at the end of the composting process, respectively. The absolute abundance of most of the ARGs in the CK+Biochar treatment was lower than that in the CK treatment during composting, and the ARGs removal mainly occurred in the early (1–14 days) and later (28–50 days) stages. Biochar addition reduced the absolute abundance of MGEs (*intI1*, *intI2*) in the compost product, and most of the ARGs had a significant positive correlation with MGEs. Network analysis and redundancy analysis showed that ARGs and MGEs occurred in various host bacteria (*Firmicutes*, *Actinobacteria*, *Bacteroidetes*, *Proteobacteria*, and *Halanaerobiaeota*), and that DTN and NH_4_^+^-N are the main factors regulating the changes in bacterial communities, antibiotics, ARGs, and MGEs during composting. Moreover, MGEs contributed the most to the variation in ARGs. In summary, biochar addition during composting accelerated antibiotics removal and inhibited accumulation and transmission of ARGs. The results of this study could provide theoretical and technical support for biochar application for antibiotics and ARGs removal during livestock and poultry manure composting.

## 1 Introduction

In the past decade, livestock and poultry manure production in China exceeded 20 billion tons, and the proportion of pig manure production surpassed 30% (Wang et al., 2021a). Antibiotics are widely used in livestock and poultry farming to promote animal growth and disease prevention (Looft et al., 2012), and in China, 97,000 tons of antibiotics are used every year. The amount of some antibiotics (fluoroquinolones, macrolides, etc.) used in pig farming is more than 50% of the total use of certain antibiotics in the farming industry (Zhang et al., 2015). However, antibiotics are incompletely absorbed in animal tissues, with 30%–90% of antibiotics excreted with animal feces and urine (Heuer et al., 2011; Cheng et al., 2019). The residual concentration of tetracycline (TC) in pig manure was noted to be the highest (Zhang et al., 2013). The discharged antibiotics may impose selective pressure on microorganisms in the environment, thereby inducing the production of antibiotic-resistant microorganisms as well as antibiotic resistance genes (ARGs) (Wei et al., 2020). Previous study had detected all known types of ARGs in pig manures from some pig farms (Zhu et al., 2013). These manures are often used as organic fertilizers in farmland, resulting in antibiotics and ARGs entering the soil and penetrating into the deeper layers of the soil over time, and even into groundwater (Xie et al., 2018; Gu et al., 2019; Menz et al., 2019). The diversity and abundance of ARGs in soil have been found to significantly increase (Chen et al., 2016), especially in farmland (Mu et al., 2015), after long-term application of untreated livestock manure. Besides, ARGs spread in the environment through horizontal gene transfer (HGT) regulated by MGEs (Su et al., 2015; Zhang et al., 2017), which further threaten human health.

Microorganisms, antibiotics, ARGs, and mobile genetic elements (MGEs) are closely related, and composting has been reported to remove antibiotics and ARGs from pig manure (Li et al., 2020; Cheng et al., 2021; Wang et al., 2021c). After composting, the removal rates of tetracyclines, sulfonamides, and macrolides antibiotics have been found to reach more than 90% (Kim et al., 2012), with some antibiotics (chlortetracycline (CTC), sulfadiazine) being completely removed (Selvam et al., 2012b). Antibiotics exert selective pressure on ARGs, and some ARGs persist even after complete degradation of antibiotics (Jechalke et al., 2014a). During pig manure composting, the relative abundances of tetracycline and sulfonamide resistance genes have been found to significantly decrease (Selvam et al., 2012a). However, composting cannot remove all ARGs, and different ARGs exhibit distinct trends during the composting process. For example, at the end of pig manure composting, the absolute abundance of *tetC* was significantly higher than the initial level (Zhang et al., 2017), whereas the abundance of *tetX* remained unchanged (Wang et al., 2015). ARGs can be transmitted between different microorganisms through MGEs (Cui et al., 2020), leading to the emergence of multi-drug-resistant microorganisms. Composting can effectively remove MGEs (Gou et al., 2018; Zhu et al., 2019), thereby inhibiting HGT of ARGs and reducing multi-drug resistance of antibiotic-resistant bacteria. A large number of pathogenic microorganisms isolated from pig manure have been reported to contain ARGs (Fang et al., 2015). Continuous high temperature during composting can lead to the inactivation of pathogenic microorganisms (Qian et al., 2016b), thereby promoting ARGs removal. Some studies have reported that continuous high temperature could promote the removal of ARGs during composting (Wang et al., 2016; Xie et al., 2016; Youngquist et al., 2016), and facilitate antibiotics removal through degradation and adsorption (Arikan et al., 2007). However, some studies have indicated that the effect of high temperature on ARGs removal are not ideal (Huang et al., 2019; Wu et al., 2020). It can be seen that whether high temperature can remove ARGs is controversial. Besides, it should be noted that most of these previous studies had been conducted to explore the effects of aerobic composting on antibiotics in pig manure, and that the antibiotics had been artificially added to pig manure (Qian et al., 2016a; Song et al., 2020; Gou et al., 2021). The knowledge about the effect of composting on antibiotics and ARGs in realistic pig manure environment is still limited.

During pig manure composting, some conditioners are usually added to regulate the moisture content (MC) and C/N ratio of the pile. Corn straw is one of the effective materials that can regulate the MC and C/N ratio of the pile. Biochar is a carbon-rich material, produced by the pyrolysis of carbon-based biomass under nitrogen-rich conditions. Biochar has been applied as an additive in composting (Qiu et al., 2021). Addition of biochar makes it easier for the compost pile temperature to rise above 70°C (Czekała et al., 2016); as a result, organic matter degradation is accelerated at the thermophilic stage of composting and nitrification is enhanced at the maturity stage of composting (Sánchez-García et al., 2015). Besides, biochar can reduce nitrogen loss and gas emission during composting (Awasthi et al., 2017), accelerate the composting process, alter the number and diversity of microorganisms during composting (Huang et al., 2020), and improve the living environment of the microorganisms and enhance their activity (Sanchez-Monedero et al., 2018). Different types of biochar prepared using diverse materials can have varying degrees of effects on bacterial communities during composting (Cui et al., 2016), which may affect the changes in ARGs. Antibiotics with animal feces into the environment caused antibiotics pollution (Sarmah et al., 2006). Furthermore, it will aggravate environmental pressure, induce the formation of microbial resistance, increase the probability of pathogen resistance, accelerate the generation and dissemination of ARGs, and pose a serious threat to human health and aquaculture (Duan et al., 2017). ARGs in the environment may invade animals or human body along the food chain or contact transmission, thereby increasing the host’s resistance to antibiotics, resulting in antibiotics cannot achieve normal therapeutic effect, but also lead to difficult to treat human and animal diseases, threatening human life and health. However, the effects of biochar on antibiotics and ARGs removal during pig manure and corn straw co-composting have been rarely reported.

Accordingly, in the present study, a mixed composting system of biochar, pig manure, and corn straw was established outdoor, with a compost volume of 2.10 m × 1.30 m × 0.75 m, and the changes in the physicochemical properties, bacterial community, antibiotics, ARGs, and MGEs in the composting system and their relationships were systematically investigated. The objectives of this study were to 1) explore the effects of biochar on the physicochemical properties of compost product during composting; 2) investigate whether biochar addition has a significant effect on the removal of antibiotics and ARGs during the composting process; 3) identify potential hosts of ARGs and MGEs; and 4) determine the dominant factors affecting antibiotics and ARGs removal during composting.

## 2 Materials and methods

### 2.1 Raw materials for composting

The raw materials used for composting included pig manure, corn straw (1–2 cm), and corn-straw biochar. Pig manure and corn straw were collected from Nongshengyuan Family Farm, Jinzhong City, Shanxi Province, China. Corn-straw biochar was prepared by the pyrolysis of corn straw at 500°C for 2 h under nitrogen-rich conditions and provided by Henan Lize Environmental Protection Technology Company. The physicochemical properties of the raw materials are shown in Table 1.

**TABLE 1:** Physicochemical properties of the composting materials.

Name	Pig manure	Corn sraw	Biochar
TOC (dry weight) g/kg	401.12	496.65	365.67
TN (dry weight) g/kg	32.00	8.40	9.80
C/N	12.54	59.13	37.31
Moisture content	66.00%	6.60%	1.90%
pH	8.50	7.16	10.24
EC mS/cm	2.13	2.40	2.41

### 2.2 Experimental design and sampling

Composting experiments were performed in open air, and CK and CK+Biochar two treatments were established. For CK treatment, pig manure (fresh weight) and corn straw (fresh weight) were mixed at a ratio of 4:1, the pile weight was 375 kg, and the MC and C/N ratio were adjusted to 70% and 25, respectively. The CK+Biochar treatment was prepared in the same procedure and 10% biochar (ratio of fresh weight of biochar to total fresh weight of pig manure and corn straw) was introduced to the pile. Because the volume of the compost pile is close to the pilot scale, each treatment has only one pile in this study. Supplementary Figure S1 shows the actual pile images of the CK and CK+Biochar treatments on day 1. It must be noted that except on day 1, the MC of the compost was not adjusted throughout the entire composting process. The pile was turned over on days 1, 3, 6, 10, 14, 21, 28, 35, 42, and 50, and samples were immediately collected at multiple points from the center of the pile and separated into two parts. One part of the samples was stored at 4°C for physicochemical properties analysis, such as MC, pH, electrical conductivity (EC), total organic matter (OM), total organic carbon (TOC), dissolved total organic carbon (DOC), total nitrogen (TN), dissolved total nitrogen (DTN), NH_4_^+^-N, NO_3_^−^-N, and seed germination index (GI) analyses, while the other part of the samples collected on days 1, 6, 14, 21, 28, 42, and 50 was stored at −20°C for bacterial community, antibiotics, ARGs, and MGEs analyses. The temperature (T) of the materials at a distance of 30 cm from the surface of the pile (represents the pile temperature) was measured at the front, middle, and back of the pile by using a thermometer at 9:00 a.m. every day.

### 2.3 Analytical investigation

#### 2.3.1 Determination of physicochemical indicators

The fresh samples were dried in an oven at 105°C for 24 h to determine the MC. The pH and EC were measured using a pH meter (PHS-3C, Shanghai, China) and conductivity meter (DDS-11A, Shanghai, China), respectively. The DOC and DTN contents were ascertained using total organic carbon analyzer (multi N/C 3100, Germany), NH_4_^+^-N and NO_3_^−^-N were measured using a flow analyzer (AMS Alliance, France), and TOC and TN contents were detected using the K_2_CrO_4_ oxidation method and Kjeldahl method. The OM content was calculated from the mass loss of dry matter after 4 h of combustion in a muffle furnace at 550°C. The GI was evaluated as described using cabbage seeds. The electrically conducting property of the biochar was investigated according to the method described by Fu et al. (2021), and the pore structure of the biochar was determined using an automated specific surface area and pore analyzer (TriStar II 3020 model, United States).

#### 2.3.2 Antibiotics quantification

The concentrations of 14 antibiotics, including four tetracyclines, two sulfonamides, five fluoroquinolones, two macrolides, and one lincosamide [lincomycin (LCM)] were measured by liquid chromatography–mass spectrometry (LC-MS 8030, Japan). The compost samples were pretreated using the method described by Wang et al. 2021b. The chromatographic conditions were as follows: Shim-pack GIST C18 column (100 mm × 2.1 mm, 2 µm); flow rate, 0.3 ml/min; injection volume, 5 μl; column temperature, 40°C; operation time, 17 min; mobile phase A, 0.2% formic acid-water; and mobile phase B, acetonitrile. The gradient washing procedure is shown in Supplementary Table S1. The mass spectrometry conditions were as follows: ionization mode, ESI positive ion mode; scanning mode, multiple reaction monitoring (MRM); collision gas, argon; atomization gas, 3 L/min nitrogen; dry gas, 15 L/min nitrogen; heating module temperature, 400°C; and DL temperature, 250°C. The antibiotics were quantified with calibration standards, and the average recoveries of tetracyclines, fluoroquinolones, macrolides, sulfadiazine (SD), sulfamethazine (SMR) and LCM in the piles were 90.83 ± 6.12%, 94.28 ± 10.45%, 70.50 ± 21.43%, 73.07%, 92.06% and 103.21%, respectively.

#### 2.3.3 DNA extraction and quantification of antibiotic resistance genes and mobile genetic elements

The DNA was extracted using rapid DNA extraction kit (TIANNAMP DNA Kit, TIANGEN, China) according to the manufacturer’s instructions. A total of 20 ARGs (*tetA*, *tetB*, *tetC*, *tetG*, *tetM*, *tetO*, *tetQ*, *tetW*, *tetX*, *tetZ*, sul1, sul2, *gyrA*, *qnrS*, *ermB*, *ermC*, *ermF*, *ermT*, *mefA*, *mphA*) and two MGEs (*intI1*, *intI2*) were quantified by PCR using StepOnePlus™ real-time PCR system (Thermo, United States). The primer sequences used for the amplification of ARGs are presented in Supplementary Table S2. The thermal cycling steps for quantitative PCR (qPCR) amplification were employed as described previously (Yin et al., 2017), and the absolute abundance of ARGs and MGEs was defined as the copy number of ARG or MGE per unit of the composting sample (copies/g).

#### 2.3.4 High-throughput sequencing for bacterial communities analysis

For the analysis of the bacterial communities in the composting system, the V3-V4 region of the 16S rRNA gene was amplified by PCR using the primers 341F/806R. The PCR amplification products were detected by 2% agarose gel electrophoresis, and the target fragment was recovered using the gel recovery kit (Qiagen). The purified PCR product was quantified by Qbit fluorescence quantification system using TruSeq^®^DNA PCR-free sample preparation kit. The amplified products were sequenced with Illumina MiSeq-PE300 sequencing platform. All the raw sequence data have been deposited in the NCBI SRA database (Accession number: PRJNA846099).

### 2.4 Data analysis

The data obtained in this study were analyzed using Microsoft Excel 2016 and SPSS version 25.0 software. The abundance heat map of the top 35 microorganisms at the genus level was plotted using TB tools. Network depictions were constructed using Cytoscape 3.9.0. Redundancy analysis (RDA) was performed using CANOCO 5.0, and structural equation model (SEM) was established using Amos Graphics.

## 3 Results and discussion

### 3.1 Changes in OM, temperature, MC, pH, and EC during composting with and without biochar

**FIGURE 1:**
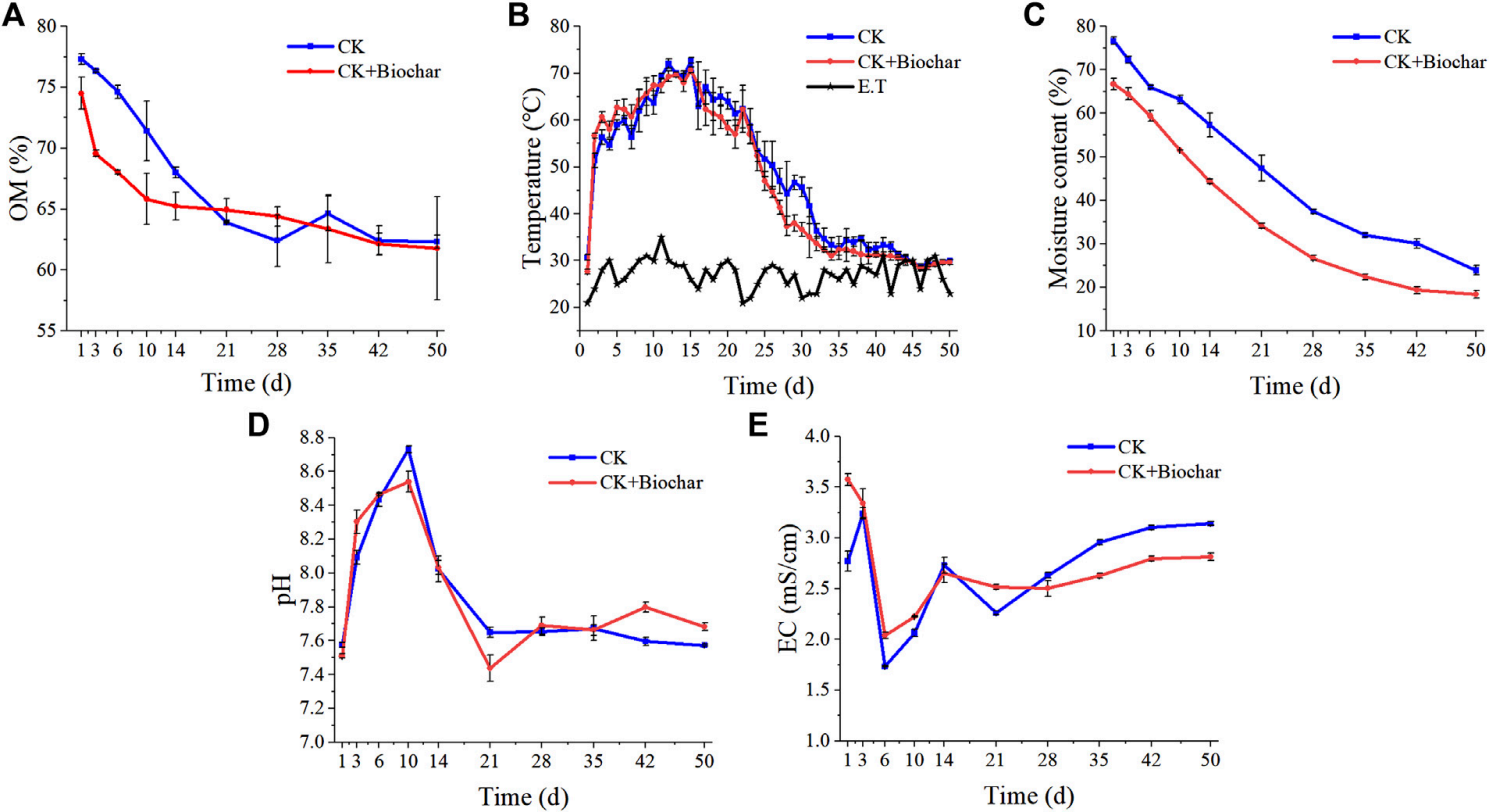
Changes in OM **(A)**, temperature **(B)**, MC **(C)**, pH **(D)**, and EC **(E)** during composting.

As shown in Figure 1A, the OM content in the CK and CK+Biochar treatments presented a downward trend during composting process. Compared with the CK treatment, the OM degradation rate in the CK+Biochar treatment was faster during 1–10 days, which was owing to the unique surface structure of biochar that provided a suitable environment for microbial activities and promoted decomposition and metabolism of OM (Sánchez-García et al., 2015). Most of the microorganisms that degrade OM are aerobic, and the biochar addition improved the aeration conditions during the composting process and facilitated contact between microorganisms and oxygen. The biochar used in the present study can conduct electricity (Supplementary Figure S2A), which could accelerate the electron transfer in the oxidation process of OM, thereby promoting OM degradation.

During composting, the temperatures of both the CK and CK+Biochar treatments showed similar trends with obvious warming, high-temperature, and cooling period (Figure 1B). However, the high temperature period (>55°C for 22 days) in the CK+Biochar treatment was longer than that noted in the CK treatment (>55°C for 20 days), which is consistent with the results of a previous study (Awasthi et al., 2020). The temperature increased above 60°C on days 3 and 8 in the CK+Biochar and CK treatments, respectively, which could be attributed to the additional nutrients and suitable living environment provided by the biochar for microbial activities, facilitating OM degradation and release of heat (Wang et al., 2013). However, the temperature of the CK and CK+Biochar treatments decreased below 40°C on days 32 and 28, respectively. Nevertheless, both the CK and CK+Biochar treatments in the present study fulfilled the safety regulations in “Sanitation of manure without harm,” which require the pile temperature to be >50°C for at least 10 days.

During composting, the MC of the CK and CK+Biochar treatments showed a downward trend (Figure 1C), exhibiting 68.78% and 72.36% decrease at the end of composting, respectively. The presence of biochar can accelerate water loss from the pile, which might be owing to the increase in the porosity of the pile (Liu et al., 2017) and optimization of the pore structure, promoting air circulation in the pile and accelerating evaporation of water (Iqbal et al., 2010). Although the increase in temperature was more rapid in the CK+Biochar treatment, the decrease in MC was slower, when compared with those noted in the CK treatment, during the early stage of composting (days 1–6), implying that temperature increase was not the main reason for moisture loss from the pile.

The pH change during composting was demonstrated in Figure 1D. The pH of both CK and CK+Biochar treatments first exhibited an increasing trend, followed by a decreasing trend, and finally stabilized. The pH values increased up to 8.73 and 8.54 in the CK and CK+Biochar treatments, respectively, on day 10 of the composting process. The volatile emission of NH_3_ was predominant during the early stage of composting (Mao et al., 2018; Ravindran et al., 2019), leading to an increase in the pH value. When compared with the initial pH value, the pH at the end of the composting process was not significantly different in both CK and CK+Biochar treatments, suggesting that the effect of biochar was not significant on pH variation before and after composting.

At the end of the composting process, the EC of both CK and CK+Biochar treatments was lower than 4 mS/cm (Figure 1E), indicating that the final compost product in both the treatments was not phytotoxic (Mao et al., 2018). In the CK treatment, the rapid decline in MC during the first 3 days may be the main reason for the slight increase in EC, which was 3.14 mS/cm higher than the initial value. In contrast, the EC of the CK+Biochar treatment was 2.81 mS/cm lower than the initial value. These results indicated that addition of biochar decreased EC and enhanced the quality of the final compost products.

### 3.2 Changes in DOC, DTN, NH_4_^+^-N, and NO_3_^−^-N during composting with and without biochar and GI of the compost products

**FIGURE 2:**
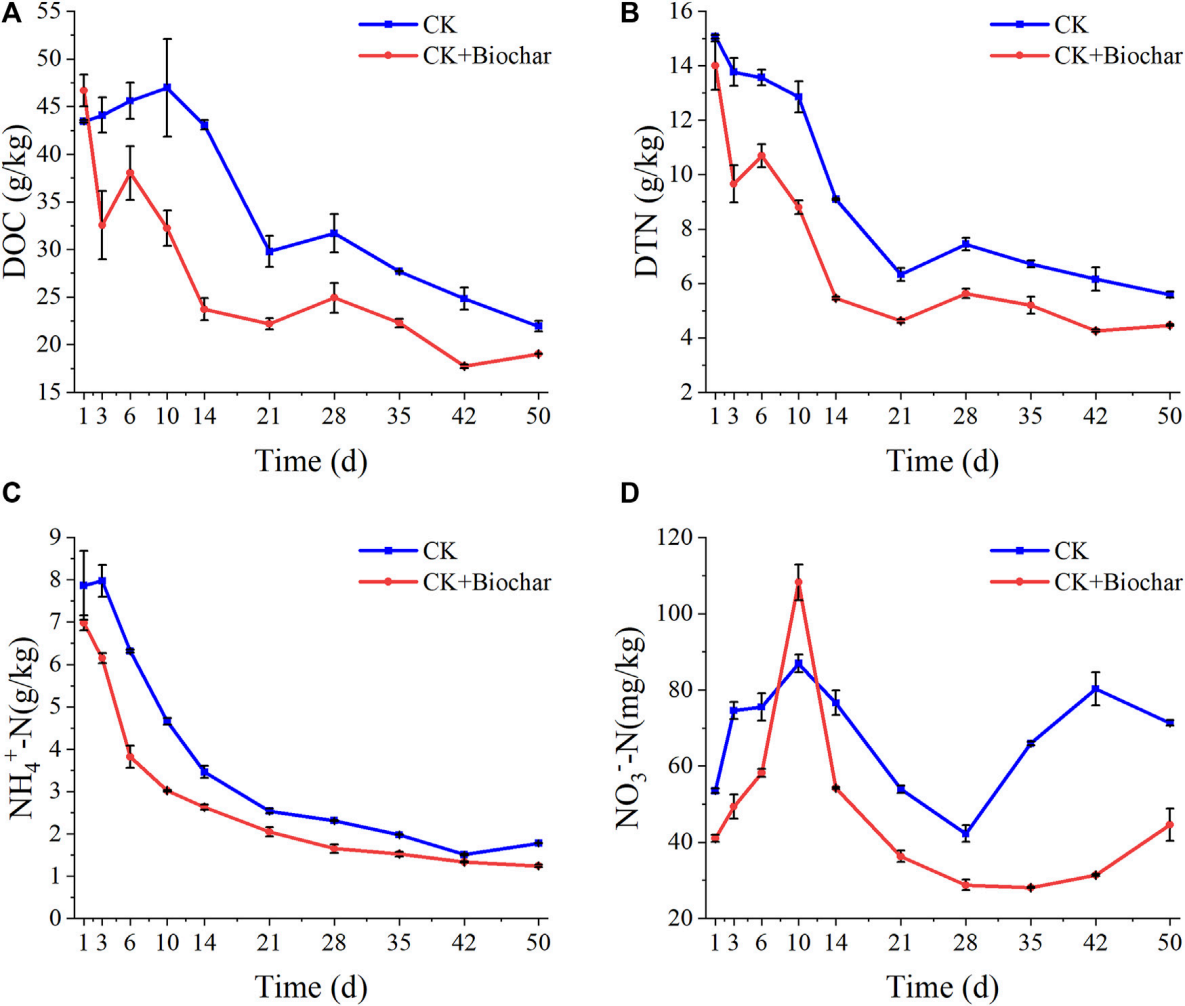
Changes in DOC **(A)**, DTN **(B)**, NH_4_^+^-N **(C)**, and NO_3_^−^-N **(D)** during composting.

The DOC content in the CK and CK+Biochar treatments showed the opposite trend in the early stage of composting (Figure 2A). The rapid decrease in the DOC content in the CK+Biochar treatment could mainly be attributed to the quick rise in temperature resulting in an increase in microbial activity (Khan et al., 2014). After 10 days of composting, the DOC content in the CK treatment started to decrease and the microbial utilization of water-soluble organic matter accelerated, probably owing to the increased activity of thermophilic microorganisms caused by high temperatures at this stage. At the beginning of the composting, the DTN content in the CK+Biochar treatment decreased significantly faster than that in the CK treatment (Figure 2B), which was owing to the rapid increase in the pile temperature of the CK+Biochar treatment. After 10 days of composting, the DTN content in the CK treatment decreased faster, which was owing to the increased activity of thermophilic microorganisms at this stage. At the end of the composting process, the DOC content decreased by 49.54% and 59.22%, and the DTN content declined by 62.94% and 68.13% in the CK and CK+Biochar treatments, respectively. These results indicated that biochar facilitated the utilization of DOC and DTN by microorganisms.

In the present study, the compost substrate contained a large number of organic nitrogen compounds, which generated NH_4_^+^-N under the action of microorganisms, leading to the increase in the NH_4_^+^-N content in the early stage of composting. As shown in Figure 2C, the NH_4_^+^-N content in the CK and CK+Biochar treatments exhibited a decreasing trend, which is not consistent with the previously reported results (Agyarko-Mintah et al., 2017). This may be owing to the high frequency of turning of the pile during the composting period in this study, which promoted oxygen exposure to the microorganisms. As NH_4_^+^-N is more likely to be converted to NO_3_^−^-N by microorganisms in the presence of high level of oxygen in the environment (Canfield et al., 2010), a large amount of NO_3_^−^-N was produced in both CK and CK+Biochar treatments in the early stage of composting (Figure 2D). However, addition of biochar increased the porosity of the pile and promoted air flow, resulting in the rapid conversion of NH_4_^+^-N in the CK+Biochar treatment, compared with that in the CK treatment. The NO_3_^−^-N content in both CK and CK+Biochar treatments reached the maximum level on day 10, presenting 62.34% and 163.70% increase, respectively, which indicated that biochar could facilitate the vital activity of nitrifying bacteria. This effect of biochar might be caused by the specific pore structure of the biochar and the adsorption sites on its surface, which are beneficial to the adsorption of NH_3_ and NH_4_^+^, and provide a suitable environment for the metabolic activities of nitrifying bacteria. After 10 days of composting, the NO_3_^−^-N content began to decrease after reaching its peak, possibly as a result of increase in the proportion of denitrifying bacteria, which promote the conversion of NO_3_^−^-N to N_2_ and N_2_O (Cáceres et al., 2018). Moreover, the temperature of the pile gradually increased to 70°C at this stage, which inhibited the activity of nitrifying bacteria. However, in the maturity stage of composting, the NO_3_^−^-N content in both CK and CK+Biochar treatments increased owing to the recovery of nitrifying bacterial activity as a result of decrease in temperature (Zainudin et al., 2020).

At the end of composting, the GI value was used to evaluate the final compost products. The GI values of the final compost products of the CK and CK+Biochar treatments were 106.82% and 171.88%, respectively. GI value above 80% implies that the final compost products are fully mature without phytotoxicity (Huang et al., 2006). The results of the present study showed that biochar addition increased the GI value and improved the quality of the final compost products.

### 3.3 Dynamic changes in the bacterial communities during composting

**FIGURE 3:**
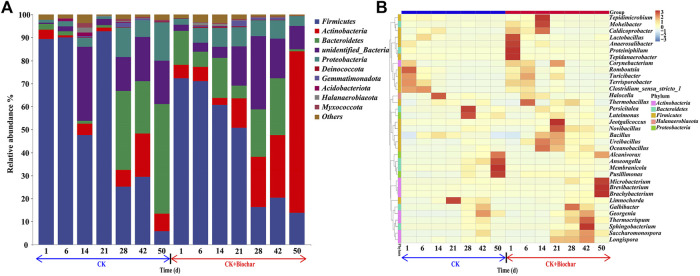
**(A)** Relative abundance of microorganisms at phylum level. **(B)** Abundance heat map of the top 35 microorganisms at genus level in different composting stages.

As shown in Figure 3A, bacterial communities evolved during the composting process. *Firmicutes*, *Actinobacteria*, *Bacteroidetes*, and *Proteobacteria* were the predominant bacterial phyla, accounting for 57.59%–97.16% of the total bacterial community, with *Firmicutes* being the major bacterial phylum in the early stage of composting. From days 1 to 21, the temperature of the CK and CK+Biochar treatments mostly remained above 50°C and *Firmicutes* were dominant in both the treatments, indicating that these bacteria could survive at high temperatures. *Firmicutes* play a key role in cellulose degradation (Lebuhn et al., 2014), and can decompose corn straw in the early stage of composting and accelerate maturation of the compost. At the genus level, *Romboutsia*, *Clostridium_sensu_stricto_1*, *Terrisporobacter*, and *Turicibacter* were predominant *Firmicutes* in both the treatments (Figure 3B). After 21 days of composting, the abundance of *Firmicutes* gradually declined, probably owing to the decrease in pile temperature, which was not suitable for the survival of most of the *Firmicutes*.

It can be observed from Figure 3A that *Actinobacteria* accounted for a relatively low proportion in the total bacterial community from days 1 to 21, because the high temperature in the early stage of composting could have inhibited the metabolic activities of *Actinobacteria*, which are thermosensitive microorganisms (Zhu et al., 2017b). However, during the maturity stage of composting, the temperature of the pile gradually decreased, and the activity of *Actinobacteria* increased, resulting in a slow increase in their proportion in the total bacterial community. At the genus level, *Brevibacterium*, *Microbacterium*, *Brachybacterium*, *Georgenia*, *Longispora*, *Saccharomonospora*, and *Corynebacterium* were the predominant *Actinobacteria* (Figure 3B), with *Brevibacterium*, *Microbacterium*, and *Brachybacterium* accounting for the highest proportion and presenting variation trend similar to that of *Actinobacteria* during composting. Furthermore, the abundance of *Actinobacteria* in the CK+Biochar treatment was higher than that in the CK treatment, with *Brevibacterium*, *Microbacterium*, and *Brachybacterium* being the dominant genera. These results demonstrated that the presence of biochar in the later composting stage stimulated the activity of *Actinobacteria*. When compared with the CK treatment, the low water content and faster water dissipation rate in the CK+Biochar treatment led to higher activity of *Actinobacteria*, because *Actinobacteria* are capable of generating spores to survive under low water condition (Zhou et al., 2019).

Throughout the composting process, the abundance of *Bacteroidetes* showed the opposite trend in the CK and CK+Biochar treatments (Figure 3A). On day 1, the abundance of *Bacteroidetes* in the CK+Biochar treatment (14.61%) was higher than that in the CK treatment (2.42%). However, in the first 6 days of composting, the abundance of *Bacteroidetes* gradually decreased in the two treatments, owing to the high pile turnover frequency. *Bacteroidetes* are anaerobic microorganisms, and oxygen supplementation can reduce their metabolic activities (López-González et al., 2021). The decrease in the abundance of *Bacteroidetes* in the CK+Biochar treatment was higher than that in the CK treatment, because biochar improved the pore structure of the pile and promoted the reaction of microorganisms to air contact. *Bacteroides*, *Moheibacter*, and *Proteiniphilum* belong to the phylum *Bacteroidetes*, and are the most sensitive to oxygen. In particular, *Moheibacter* was active during the highest temperature period (6–14 days), probably because of its thermophilic characteristic. At the end of the composting, the abundance of *Bacteroidetes* in the CK and CK+Biochar treatments was 47.56% and 0.86%, respectively, and this difference mainly occurred in the maturity stage. During the maturity stage of composting, the abundance of *Bacteroidetes* in the CK treatment increased, whereas that in the CK+Biochar treatment sharply decreased, especially in the last 8 days of composting, which was predominantly owing to the changes in the abundances of *Anseongella* and *Membranicola* (Figure 3B).

Although the abundance of *Proteobacteria* gradually increased in both CK and CK+Biochar treatments in the early stage of composting, the reproduction rate and abundance of *Proteobacteria* was higher in the CK+Biochar treatment. It has been reported that *Proteobacteria* have a key role in nitrogen transformation processes such as oxidation of ammonia in compost (Maeda et al., 2011). Thus, the NH_4_^+^-N content in the CK+Biochar treatment rapidly decreased in the early stage of composting, whereas the NO_3_^−^-N content was higher (Figure 2). In the later stage of composting, the higher abundance of *Proteobacteria* led to higher NO_3_^−^-N content in the CK treatment.

### 3.4 Changes in antibiotics contents during composting

**FIGURE 4:**
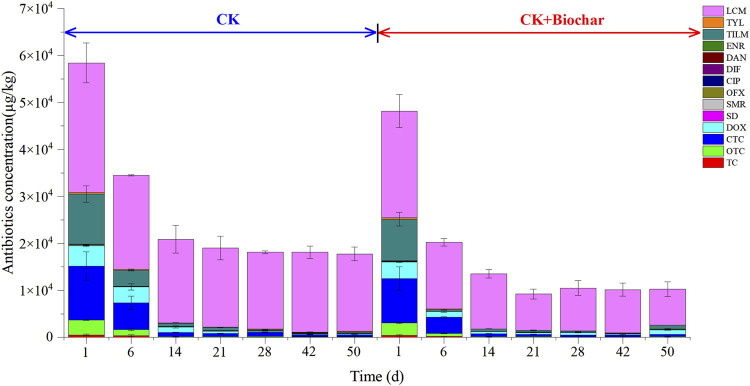
Concentration of antibiotics in different periods of composting.

Figure 4 presents the changes of tetracyclines (TC), oxytetracycline (OTC), CTC, doxycycline (DOX), sulfonamides (SD, SMR), fluoroquinolones [ofloxacin (OFX), ciprofloxacin (CIP), difloxacin (DIF), danofloxacin (DAN), enrofloxacin (ENR)], macrolides [tilmicosin (TILM), tylosin (TYL)], and lincosamides (lincomycin (LCM)) antibiotics during composting in the CK and CK+Biochar treatments. At the end of composting, the total antibiotics removal rates in the CK and CK + Biochar treatments were 69.58% and 78.67%, respectively. The antibiotics removal mainly occurred in the early stage of composting (1–14 days), and the total antibiotics removal rates in the CK and CK+Biochar treatments were 64.24% and 71.84%, respectively. At the same time, temperature had a significant negative correlation with TC, OTC, CTC, DC, SD, SMR, OFX, CIP, ENR, TYL, TILM, and LCM concentration in the first 14 days of composting (Table 2), indicating that the increase in temperature was beneficial to antibiotics removal. This finding is consistent with that reported in previous studies, which revealed that high temperature could promote the degradation of antibiotics (Arikan et al., 2007; Dorival-García et al., 2013; Liu et al., 2015). The addition of biochar accelerated the increase in pile temperature, and consequently, the antibiotics removal rate in the CK+Biochar treatment was higher in the early stage of composting.

**TABLE 2:** Pearson’s correlation coefficient between temperature and antibiotics at 1–14 days of composting.

	TC	OTC	CTC	DOX	SD	SMR	OFX	CIP	DIF	DAN	ENR	TYL	TILM	LCM
T	−0.868*	−0.967**	−0.951**	−0.837*	−0.349	−0.934**	−0.952**	−0.938**	−0.901*	−0.703	−0.869*	−0.883*	−0.967**	−0.836*

*p < 0.05, **p < 0.01.

During the first 6 days of composting, 50.93%, 74.77%, 63.85% 66.13%, and 95.82% of TC, OTC, CTC, DOX, and TILM were removed in the CK+Biochar treatment, respectively, which were higher than those noted in the CK treatment (30.53%, 58.70%, 50.79%, 21.00%, and 68.28%, respectively). Biodegradation is an important pathway for the removal of tetracyclines antibiotics during pig manure composting (Wang et al., 2015). It must be noted that tetracyclines antibiotics are easily adsorbed by the medium in the environment (Tolls, 2001; Thiele-Bruhn and Beck, 2005), and addition of biochar, which has a rich pore structure and large number of binding sites, can facilitate the adsorption of tetracyclines antibiotics on the biochar surface and pores, and enhance the degradation of tetracyclines antibiotics by microorganisms adhering to the biochar. During 1–14 days of composting, the TYL removal rates in the CK and CK+Biochar treatments were 77.08% and 94.44%, respectively. TYL and TILM are amphoteric compounds with positive charges, which can form ion bonds with negatively charged components, such as the biochar surface, during composting (Tan et al., 2020); as a result, their removal was accelerated with biochar addition. At the end of composting, the removal rates of SD, SMR, OFX, CIP, DIF, and LCM in the CK treatment were 55.88%, 77.10%, 48.65%, 52.86%, 38.21%, and 40.23%, respectively, which were lower than those noted in the CK+Biochar treatment (68.32%, 84.75%, 87.24%, 64.73%, 62.55%, and 66.40%, respectively). In the presence of organic matter, high temperatures can lead to the production of more adsorption sites, decreasing the extractable concentration of sulfonamides antibiotics (Ezzariai et al., 2018). Biochar addition can inhibit the production of CO_2_ and reduce the loss of organic matter (Liu et al., 2017), thereby promoting the removal of sulfonamides in pig manure. The molecular structure of quinolone antibiotics comprises a large number of carboxyl, amino, and aromatic rings, which are easily adsorbed by humus (Kümmerer, 2009). At the end of composting, the total humic acid contents of CK and CK + Biochar treatments increased by 24.16% (from 62.02 g/kg to 77.01 g/kg) and 47.96% (from 64.78 g/kg to 95.85 g/kg), respectively. Biochar promotes the formation of humus during composting. Biodegradation is considered to be the main mechanism of LCM degradation (Alexandrino et al., 2017; Ren et al., 2018) and biochar can adsorb LCM for more than 200 days, exhibiting strong adsorption performance (Liu et al., 2019), which facilitates biodegradation of LCM. *Bacillus*, *Saccharomonospora*, and *Georgenia* presented significant negative correlations with TC, DOX, SD, SMR, DIF, TYL, and LCM concentrations (Supplementary Figure S4), indicating that these microorganisms can degrade antibiotics. Addition of biochar improved the activity of these microorganisms, thereby promoting the degradation of antibiotics during composting. In CK and CK+Biochar treatments, the concentration of DAN increased from 28.47 to 32.00 μg/kg and 23.49 to 32.32 μg/kg, and the concentration of ENR increased from 82.50 to 105.49 μg/kg and 68.06 to 146.32 μg/kg. *Actinobacteria* is considered to be the main producer of antibiotics (D'Costa et al., 2006), and *Microbacterium* and *Brachybacterium*, which belong to the phylum *Actinobacteria,* were significantly positively correlated with DAN and ENR concentrations, and their growth and reproduction during composting led to an increase in DAN and ENR concentrations. The results of this study revealed the high efficiency of antibiotic removal from co-composting of pig manure and corn straw with biochar. The removal rates of macrolide antibiotics were higher than that of other antibiotics at the early composting stage due to the differences in the types and chemical structures of antibiotics. Moreover, microorganisms are also important factors affecting the change of antibiotic concentration during composting.

### 3.5 Dynamic variations in antibiotic resistance genes and mobile genetic elements during composting

#### 3.5.1 Changes in the absolute abundance of antibiotic resistance genes during composting

**FIGURE 5:**
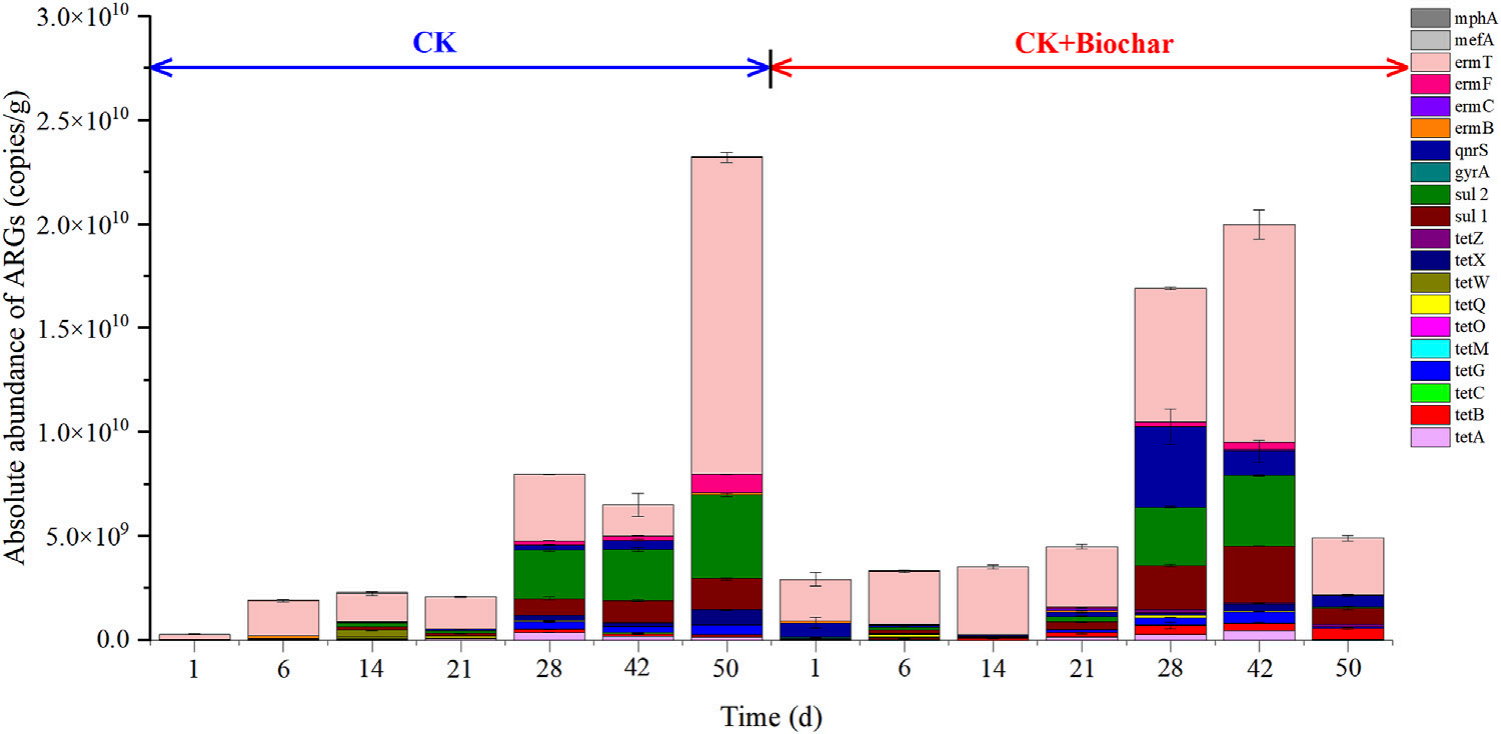
Absolute abundance of ARGs in different periods of composting.

Figure 5 shows the changes in the absolute abundance of ARGs (tetracyclines, sulfonamides, fluoroquinolones, macrolides, and lincosamides resistance genes) during composting. On day 1, biochar addition increased the absolute abundance of ARGs, similar to that reported in a previous study (Zhou et al., 2021). The reason for the increase in the absolute abundance of ARGs following biochar addition may be the higher abundance of *Bacteroidetes* and *Proteobacteria*, the major host bacteria of ARGs (Stegmann et al., 2015; Luo et al., 2017), in the CK+Biochar treatment, when compared with those in the CK treatment. However, with the progress in the composting process, the absolute abundance of the total ARGs increased by 81.24 times in the CK treatment, which was higher than that noted in the CK+Biochar treatment (0.48 times), indicating that biochar addition was beneficial to inhibit the increase in the absolute abundance of ARGs.

After 50 days of composting, the absolute abundance of tetracycline resistance genes changed in varying degrees of changes. In the CK treatment, only *tetO* abundance decreased by 49.63% after the composting process because anaerobic bacteria are the major hosts of this ARG (Wang et al., 2015), whereas the absolute abundances of the other nine tetracycline resistance genes increased at varying degrees, with *tetA* and *tetM* presenting the highest and lowest increase in abundance, respectively (3.63×10^3^ and 1.52 times, respectively). In contrast, in the CK+Biochar treatment, the absolute abundances of *tetM*, *tetO*, *tetW*, and *tetX* decreased by 92.83%, 46.30%, 24.15%, and 57.86%, respectively, whereas those of *tetA*, *tetC*, *tetG*, and *tetZ* increased by 7.8, 0.4, 11.6, and 500 times, respectively, and *tetB* and *tetQ* were undetectable after the composting process. These findings indicated that biochar addition led to higher removal of tetracycline resistance genes from the compost products.

The absolute abundances of sulfonamide resistance genes *sul1* and *sul2* increased by 3.80×10^3^ and 6.56 × 10^3^ times, respectively, in the CK treatment, and 33.95 and 4.06 times, respectively, in the CK+Biochar treatment, revealing that biochar addition to the composting system significantly decreased *sul1* and *sul2* abundances, predominantly after 42 days of composting. Similarly, Guo et al. (2021) also found that ARGs were mainly removed in the mature stage of pig manure composting.

The fluoroquinolones resistance genes, *gyrA* and *qnrS,* were not detected in the final product of the CK treatment, whereas their absolute abundances decreased by 54.02% and 22.05%, respectively, in the CK+Biochar treatment, indicating that biochar addition might have promoted the growth and reproduction of bacteria possessing *gyrA* and *qnrS.* The absolute abundance of macrolide and lincosamide resistance genes in the CK treatment increased at varying degrees at the end of the composting process, with *ermF* and *ermC* presenting the highest and lowest increase of 2.54×10^3^ and 5.12 times, respectively. In the CK+Biochar treatment, the absolute abundances of *ermB* and *mefA* decreased at the end of composting, with their removal rates reaching 90.77% and 53.51%, respectively, whereas those of *ermC*, *ermF*, *ermT*, and *mphA* increased with the progress of the composting process.

In summary, biochar addition to the composting system promoted the removal of ARGs by prolonging and maintaining the duration of high-temperature period, which resulted in the killing of some heat-sensitive bacteria possessing ARGs. As shown in Figure 5, the absolute abundance of ARGs in the CK and CK+Biochar treatments gradually increased after the start of composting, which could be owing to elevated temperatures facilitating increase in the activities of *Firmicutes* that carry and spread ARGs (Huerta et al., 2013). Addition of biochar promoted ARGs removal in the later stages of composting (42–50 days), due to the decrease in the relative abundance of *Bacteroidetes* and *Proteobacteria*. It has been reported that additional carbon sources can lead to a transient increase in the abundance of ARGs, followed by a decrease (Duan et al., 2018), and can degrade antibiotics, eliminate pathogens, and reduce the diversity and relative abundance of ARGs.

#### 3.5.2 Changes in the absolute abundance of mobile genetic elements during composting

As HGT is considered to be an important mechanism for the spread and proliferation of ARGs in various environmental media (Thomas and Nielsen, 2005), the present study explored the changes in the absolute abundances of *intI1* and *intI2* during pig manure composting (Table 3). At the end of the composting process, the absolute abundance of *intI1* in the CK and CK+Biochar treatments increased by 3.48 × 10^7^ and 6.69 × 10^4^ times, respectively. It has been reported that biochar addition could inhibit the increase in *intI1* abundance during composting (Li et al., 2017). The absolute abundance of *intI2* showed the opposite trend, presenting an increase of 1.51 × 10^5^ times in the CK treatment and a decrease of 34.75% in the CK + Biochar treatment. Biochar contains a large number of micropores (Supplementary Figure S2B), which can enhance the spatial distance between microorganisms and reduce the possibility of mutual contact between microorganisms. As HGT of ARGs mainly occurs through interaction between microorganisms (Martínez et al., 2015), biochar could reduce the occurrence of HGT, resulting in the removal of ARGs. In the present study, a significant positive correlation was observed between *intI1* and *intI2* and most of the ARGs during the composting process, indicating that *intI1* and *intI2* played an important role in the spread of ARGs.

**TABLE 3:** Absolute abundance of MGEs in different periods of composting.

MGEs	int I1 (copies/g)		int I2 (copies/g)	
Time (d)/Treatment	CK	CK + Biochar	CK	CK + Biochar
1	1.08E+05 ± 1.50E+04	2.96E+05 ± 2.74E+04	1.18E+05 ± 1.81E+04	8.49E+05 ± 1.34E+05
6	9.08E+09 ± 1.81E+09	2.60E+11 ± 1.99E+10	5.22E+05 ± 5.97E+04	3.39E+06 ± 8.49E+05
14	1.38E+10 ± 3.27E+09	1.37E+08 ± 3.20E+07	1.33E+06 ± 5.89E+05	2.37E+06 ± 4.84E+05
21	1.68E+10 ± 5.62E+09	2.28E+10 ± 3.52E+09	1.51E+06 ± 5.94E+05	5.33E+06 ± 1.01E+06
28	2.11E+10 ± 5.58E+08	4.21E+11 ± 1.94E+10	2.25E+06 ± 7.94E+05	1.40E+08 ± 5.13E+06
42	5.31E+06 ± 6.57E+06	8.08E+11 ± 1.12E+11	0.00E+00	4.21E+07 ± 9.03E+05
50	3.76E+12 ± 2.62E+11	1.98E+10 ± 4.36E+09	1.78E+08 ± 1.43E+07	5.54E+05 ± 3.00E+03

### 3.6 Relationship between potential host bacteria and antibiotic resistance genes and mobile genetic elements

**FIGURE 6:**
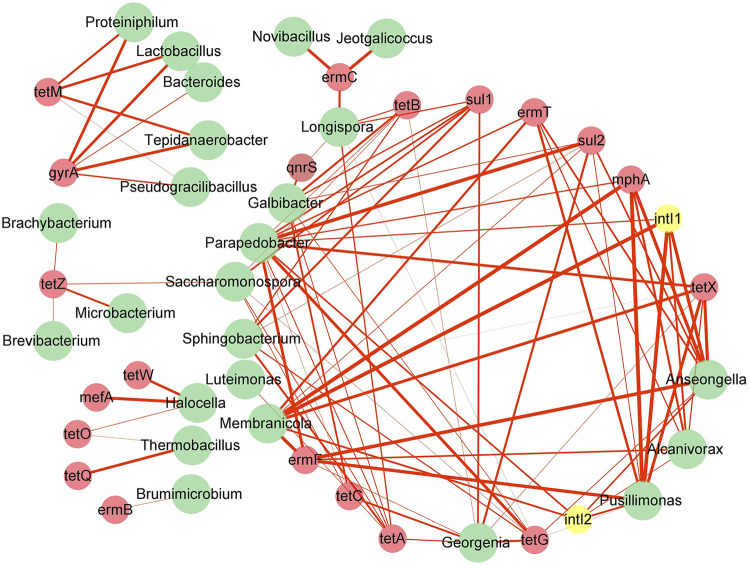
Network analysis based on co-occurrence of ARGs, MGEs, and their potential host bacteria. The red node represents ARGs, yellow node denotes MGEs, green node indicates bacteria at the genus level, and red line implies significant (*p* < 0.05) positive correlation (*r* > 0). The thicker the line is, the stronger is the positive correlation.

Network analysis was used in this study to determine the potential host bacteria of ARGs and MGEs (Figure 6). A total of 24 genus-level bacteria were significantly associated with 20 ARGs and 2 MGEs (*p* < 0.05). It has been reported that a significant positive correlation between ARGs and bacterial communities could provide new insights into the potential host bacteria of ARGs (Zhu et al., 2017a). A total of 24 bacterial genera, belonging to *Firmicutes*, *Actinobacteria*, *Bacteroidetes*, *Proteobacteria*, and *Halanaerobiaeota,* were found to be potential host bacteria of ARGs and MGEs. *Lactobacillus* was observed to be a potential host of *gyrA* and t*etM*, and the removal of *gyrA* and *tetM* during the composting process could be attributed to the decrease in the abundance of *Lactobacillus*, which is consistent with the findings of previous studies suggesting that *Lactobacillus* is a potential host of ARGs (Bonham et al., 2017). As the host bacteria of *tetZ* are *Brachybacterium*, *Saccharomonospora*, *Microbacterium*, and *Brevibacterium*, the increase in the abundance of *tetZ* was mainly owing to the proliferation of these four bacteria. These results showed that ARGs existed in various possible host bacteria, which is consistent with the previously reported findings that indicated that the succession of bacterial communities led to variations in ARGs abundance (Sun et al., 2018). Furthermore, *Parapedobacter*, *Anseongella*, *Georgenia*, *Pusillimonas*, *Galbibacter*, *Membranicola*, *Alcanivorax*, *Longispora*, *Saccharomonospora*, and *Sphingobacterium* were noted to carry more ARGs and MGEs (at least five or more ARGs and MEGs), with *Parapedobacter* possessing 12 kinds of ARGs and MGEs. Besides, the hosts of some ARGs were not only one type of bacteria, but a variety of bacteria; for instance, *tetG* was found to have as many as nine potential host bacteria. The correlation between ARGs and different potential hosts varied, which was mainly related to the spread of ARGs among bacteria through HGT (Forsberg et al., 2014). The use of multiple antibiotics can lead to the emergence of multi-resistant microorganisms.

Network analysis showed that *intI1* and *intI2* have the same potential hosts, namely, *Parapedobacter, Alcanivorax*, *Pusillimonas*, *Anseongella*, and *Membranicola* (Figure 6). These bacteria were significantly associated with a variety of ARGs (*p* < 0.05) and were the hub of ARGs for HGT, consistent with that reported in a previous study suggesting that MGEs are associated with multidrug resistance genes (Jechalke et al., 2014b). The variations in the abundances of *intI1* and *intI2* during the composting process were strongly affected by bacterial communities, which also supported the view that the changes in the bacterial communities could affect the proliferation and propagation of ARGs.

### 3.7 Relationships among environmental factors, antibiotics, bacterial community, antibiotic resistance genes, and mobile genetic elements

**FIGURE 7:**
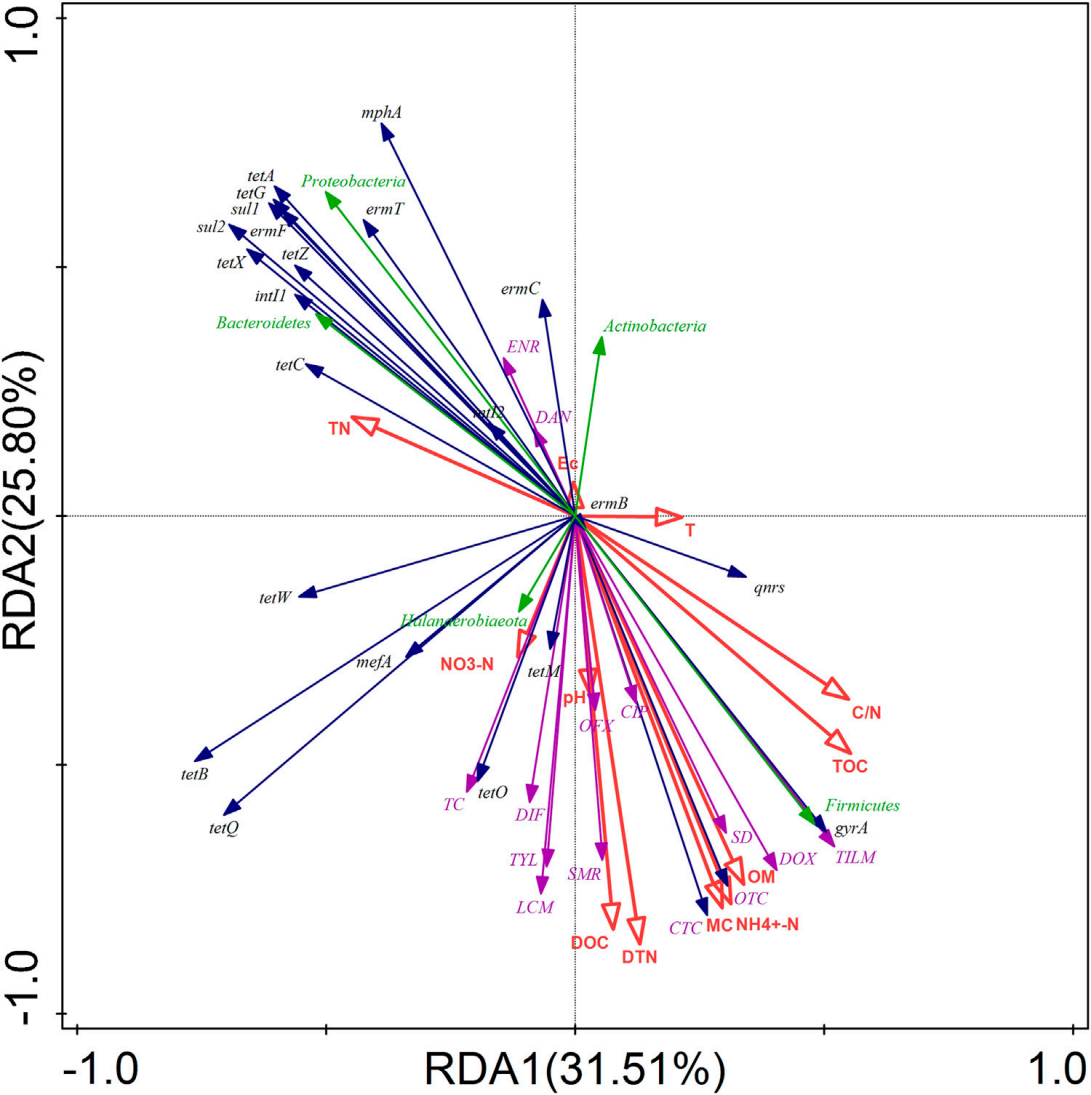
RDA of the relationship between environmental factors (red arrows) and ARGs, MGEs (blue arrows), antibiotics (purple arrows), and phylum-level bacteria (green arrows) during composting.

RDA was used to determine the effects of environmental factors on antibiotics, ARGs, MGEs, and bacterial communities during the composting process (Figure 7). The first two axes (RDA1 and RDA2) explained 57.31% of the changes in the bacterial communities, antibiotics, ARGs, and MGEs during composting. Among the environmental factors selected in this study, DTN and NH_4_^+^-N presented the highest contribution of 22.60% and 21.80%, respectively, and showed significant correlation with *Firmicutes* and *Proteobacteria* (*p* < 0.05). This result is consistent with the findings of a previous study that demonstrated that nitrogen content is closely related to microbial growth (Chen et al., 2014). Furthermore, a significant positive correlation was observed between DTN and NH_4_^+^-N contents and tetracyclines antibiotics, sulfonamides antibiotics, OFX, CIP, DIF, macrolides antibiotics, and LCM, indicating that biochar addition facilitated antibiotics removal from swine manure by promoting DTN utilization and NH_4_^+^-N transformation by microorganisms. In contrast, a significant negative correlation was noted between DTN and *sul1* as well as NH_4_^+^-N and *sul1*, *sul2*, *tetA*, and *tetG* (Supplementary Figure S3), which could explain the observed increase in the absolute abundance of *sul1*, *sul2*, *tetA*, and *tetG* during the composting process. Furthermore, OM, MC, DOC, TOC, and C/N exhibited significant correlations with some antibiotics, ARGs, and microorganisms, indicating that the changes in the environmental factors were critical to the composting process (Supplementary Figure S3).

**FIGURE 8:**
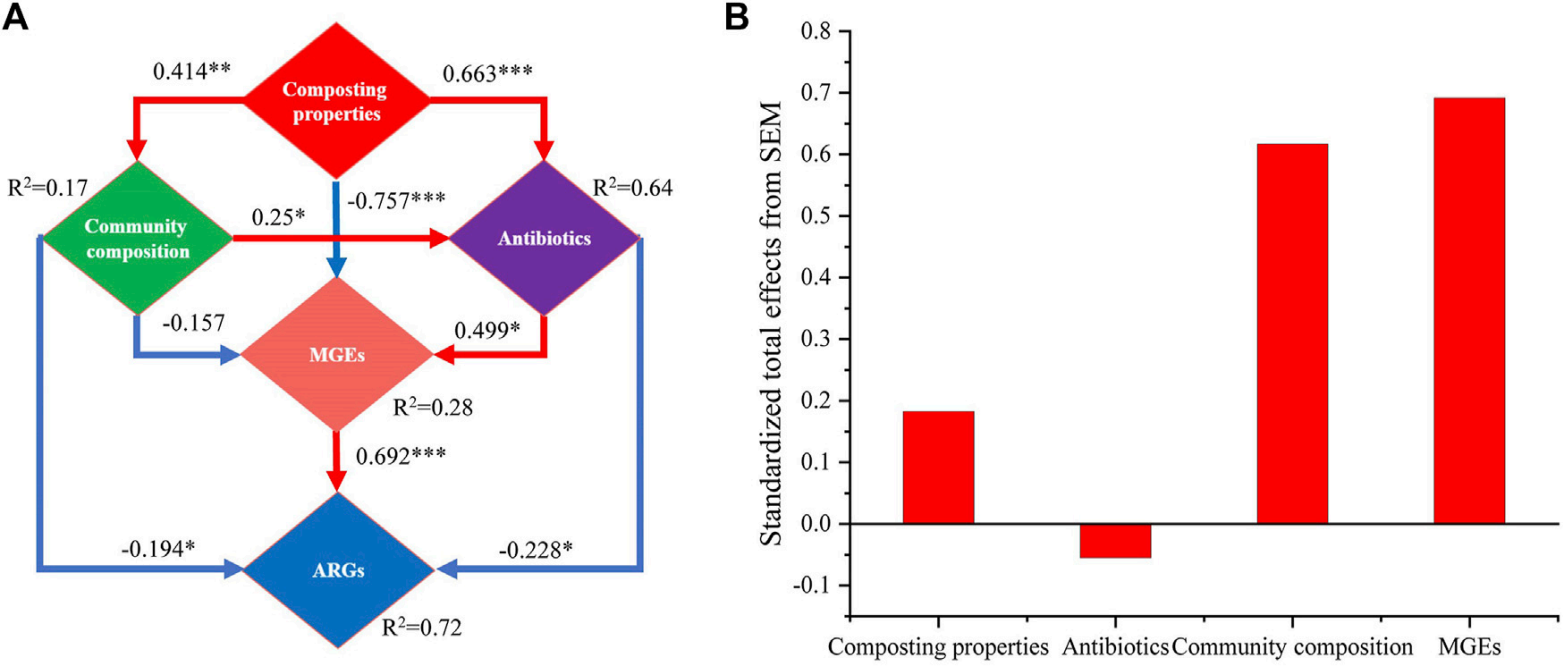
**(A)** SEM reflecting direct and indirect relationships between compost physicochemical properties, antibiotics, bacterial communities, MGEs, and ARGs. *GFI* = 0.953. **(B)** Total effect of standardization estimated based on SEM. Red arrow indicates positive correlation and blue arrow denotes negative correlation. *p < 0.05, **p < 0.01, ***p < 0.001.

To further explore how antibiotics and ARGs were affected by other driving factors in the composting process, the direct and indirect relationships between different variables were examined by establishing a SEM (Figure 8). The results showed that the composting properties (*λ* = 0.663, *p* < 0.001) and community composition (*λ* = 0.250, *p* < 0.05) had significant effects on the variations in the antibiotics levels during composting. The composting properties were the dominant factors that determined the degradation status of antibiotics. Moreover, the variations in the ARGs abundances during composting were mainly influenced by antibiotics, bacterial community composition, and MGEs, with MGEs (*λ* = 0.692, *p* < 0.001) being the highest contributor to the changes in ARGs, followed by antibiotics (*λ* = −0.228, *p* < 0.05) and bacterial community composition (*λ* = −0.194, *p* < 0.05). This finding is consistent with those reported in previous studies that demonstrated that the contribution of MGEs to changes in ARGs was higher than that of bacterial community composition during composting (Ma et al., 2017; Wu et al., 2017). The effect of composting properties on ARGs was mainly indirect *via* the influence of antibiotics (*λ* = 0.663, *p* < 0.001), bacterial community composition (*λ* = 0.414, *p* < 0.01), and HGT dominated by MGEs (*λ* = −0.757, *p* < 0.01) on the changes in ARGs during composting. Moreover, addition of biochar, directly and indirectly, affected the changes in antibiotics and ARGs during composting by altering the composting properties.

## 4 Conclusion

During pig manure and corn straw co-composting, biochar addition can: 1) accelerate the pile temperature, prolong the high-temperature period (>55°C), and reduce the phytotoxicity of the compost product; 2) promote the transformation of NH_4_^+^-N and formation of NO_3_^−^-N in the early stage of composting, and improve the DOC and DTN utilization rates; 3) accelerate the degradation of OM and water dissipation, and improved the GI value of the final composting product; and 4) quicken the degradation of antibiotics at the early stage of composting, change the bacterial community structure during composting, and promote the removal of ARGs and MGEs. Temperature was closely related to the degradation of antibiotics in the early stage of composting, and the composting properties affected the removal of antibiotics during composting. The bacterial communities and MGEs were the main factors driving the variations in ARGs, and biochar inhibited HGT of ARGs by reducing the abundance of MGEs. Thus, biochar addition can reduce the environmental risk of antibiotics and ARGs in the compost product.

## Data Availability

The sequences data reported in this study have been deposited in NCBI SRA with the accession number PRJNA846099 (https://www.ncbi.nlm.nih.gov/sra/PRJNA846099).
