# Supplementary material for: Effect of biochar on antibiotics and antibiotic resistance genes variations during co-composting of pig manure and corn straw
Source: Front Bioeng Biotechnol. 2022 Jul 22;10:960476. doi: 10.3389/fbioe.2022.960476 (PMC9377313; doi:10.3389/fbioe.2022.960476)
Supplement: Supplementary file 1 [file DataSheet1.docx]

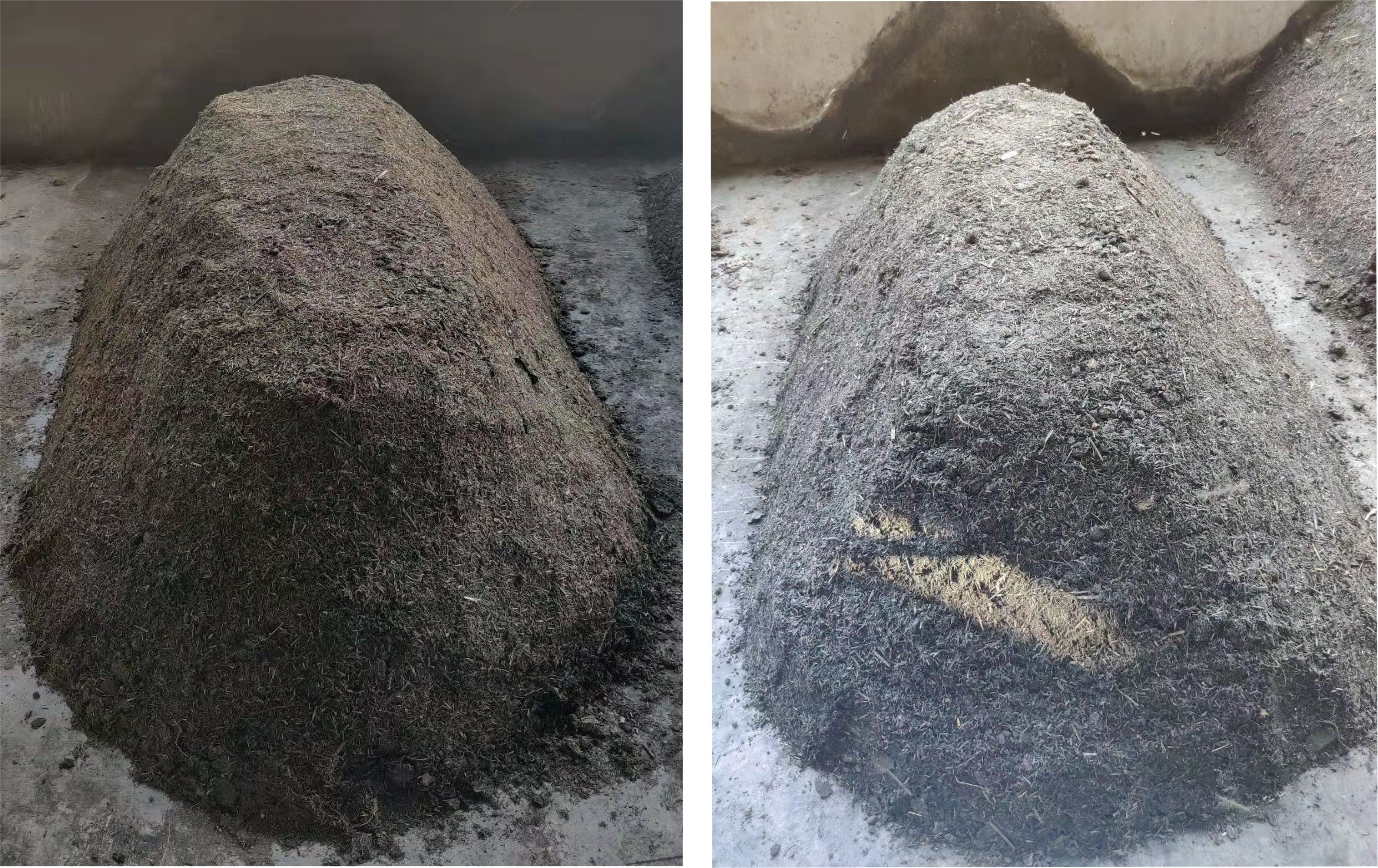


**FIGURE S1** The pile images of CK (left) and CB + Biochar treatment (right) at 1 d.

**TABLE S1** Gradient wash procedure for high performance liquid.

| Time/min | 0 | 3 | 10 | 13 | 14 | 17 |
| --- | --- | --- | --- | --- | --- | --- |
| A (%) | 90 | 90 | 65 | 10 | 90 | 90 |
| B (%) | 10 | 10 | 35 | 90 | 10 | 10 |

**TABLE S2** Primer sequences used for amplification of antibiotic resistance genes.

| Gene | F | R | |
| --- | --- | --- | --- |
| 16s | GGGTTGCGCTCGTTGC | ATGGYTGTCGTCAGCTCGTG | |
| tetA | CTCACCAGCCTGACCTCGAT | CACGTTGTTATAGAAGCCGCATAG | |
| tetB | AGTGCGCTTTGGATGCTGTA | AGCCCCAGTAGCTCCTGTGA | |
| tetC | ACTGGTAAGGTAAACGCCATTGTC | ATGCATAAACCAGCCATTGAGTAAG | |
| tetG | TCAACCATTGCCGATTCGA | TGGCCCGGCAATCATG | |
| tetM | CATCATAGACACGCCAGGACATAT | CGCCATCTTTTGCAGAAATCA | |
| tetO | ATGTGGATACTACAACGCATGAGATT | TGCCTCCACATGATATTTTTCCT | |
| tetQ | CGCCTCAGAAGTAAGTTCATACACTAAG | TCGTTCATGCGGATATTATCAGAAT | |
| tetW | ATGAACATTCCCACCGTTATCTTT | ATATCGGCGGAGAGCTTATCC | |
| tetX | AAATTTGTTACCGACACGGAAGTT | CATAGCTGAAAAAATCCAGGACAGTT | |
| tetZ | CCTTCTCGACCAGGTCGG | ACCCACAGCGTGTCCGTC | |
| sul1 | CACCGGAAACATCGCTGCA | AAGTTCCGCCGCAAGGCT | |
| sul2 | GTCAAAGAACGCCGCAATGT | TCATCTGCCAAACTCGTCGTTA | |
| gyrA | CCAACAATGACCGACATCGC | GCGGTTAGATGAGCGACCTT | |
| qnrS | GTGAGTAATCGTATGTACTTTTGC | AAACACCTCGACTTAAGTCT | |
| ermB | TAAAGGGCATTTAACGACGAAA | TTTATACCTCTGTTTGTTAGGGAATTGAA | |
| ermC | TTTGAAATCGGCTCAGGAAAA | ATGGTCTATTTCAATGGCAGTTACG | |
| ermF | CAGCTTTGGTTGAACATTTACGAA | AAATTCCTAAAATCACAACCGACAA | |
| ermT | CATATAAATGAAATTTTGAG | ACGATTTGTATTTAGCAACC | |
| mefA | CCGTAGCATTGGAACAGCTTTT | AAACGGAGTATAAGAGTGCTGCAA | |
| mphA | CTGACGCGCTCCGTGTT | GGTGGTGCATGGCGATCT | |
| intI1 | CGAACGAGTGGCGGAGGGTG | TACCCGAGAGCTTGGCACCCA |  |
| intI2 | TGCTTTTCCCACCCTTACC | GACGGCTACCCTCTGTTATCTC |  |


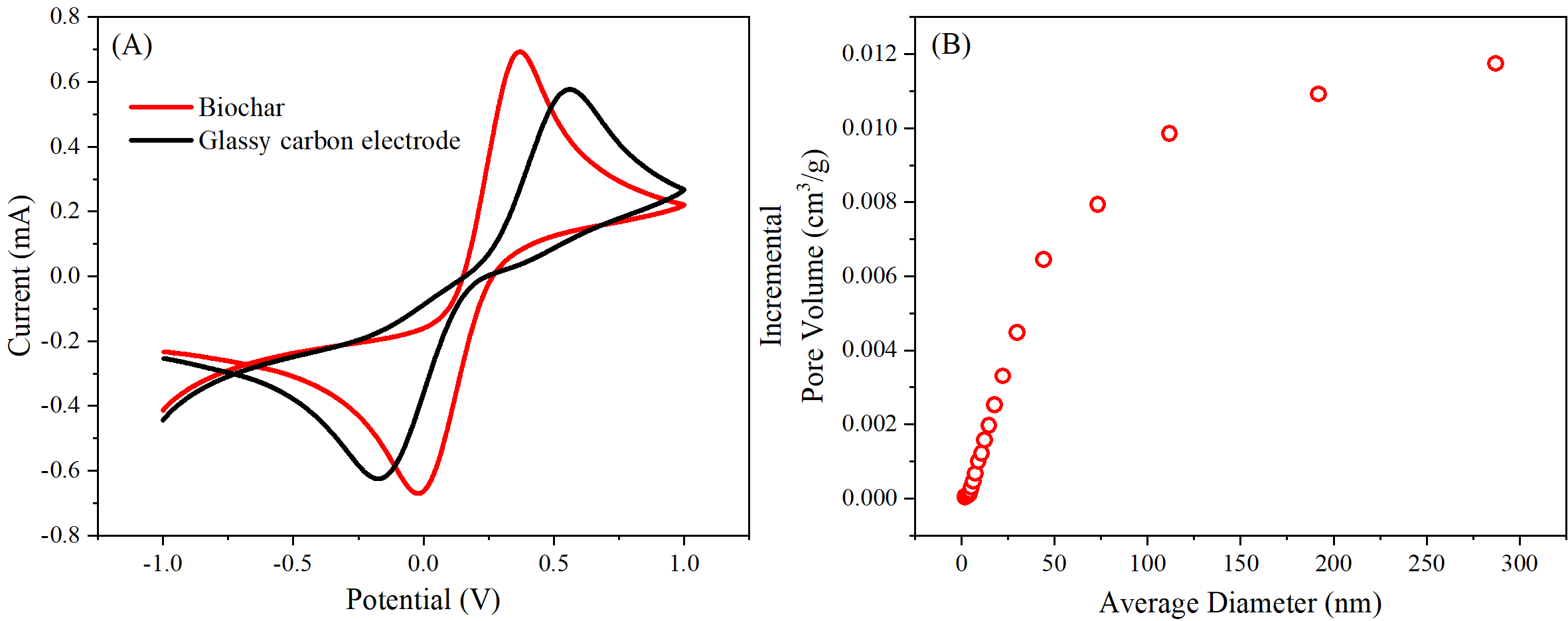


**FIGURE S2** (A) Cyclic voltammogram characteristic curves of biochar and glassy carbon electrode. (B) Incremental pore volume of biochar.


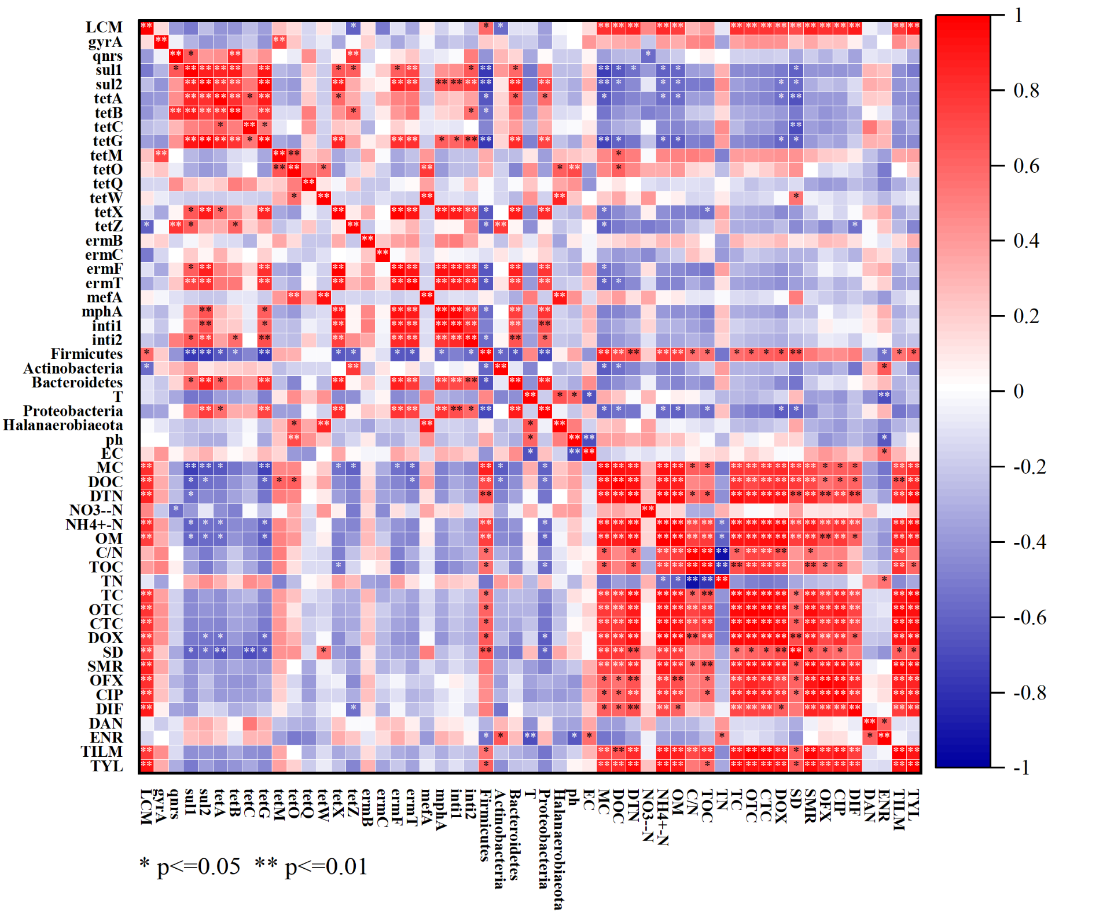


**FIGURE S3** Pearson's correlation coefficients between environmental factors, antibiotics, phylum-level microorganisms, ARGs, and MGEs.


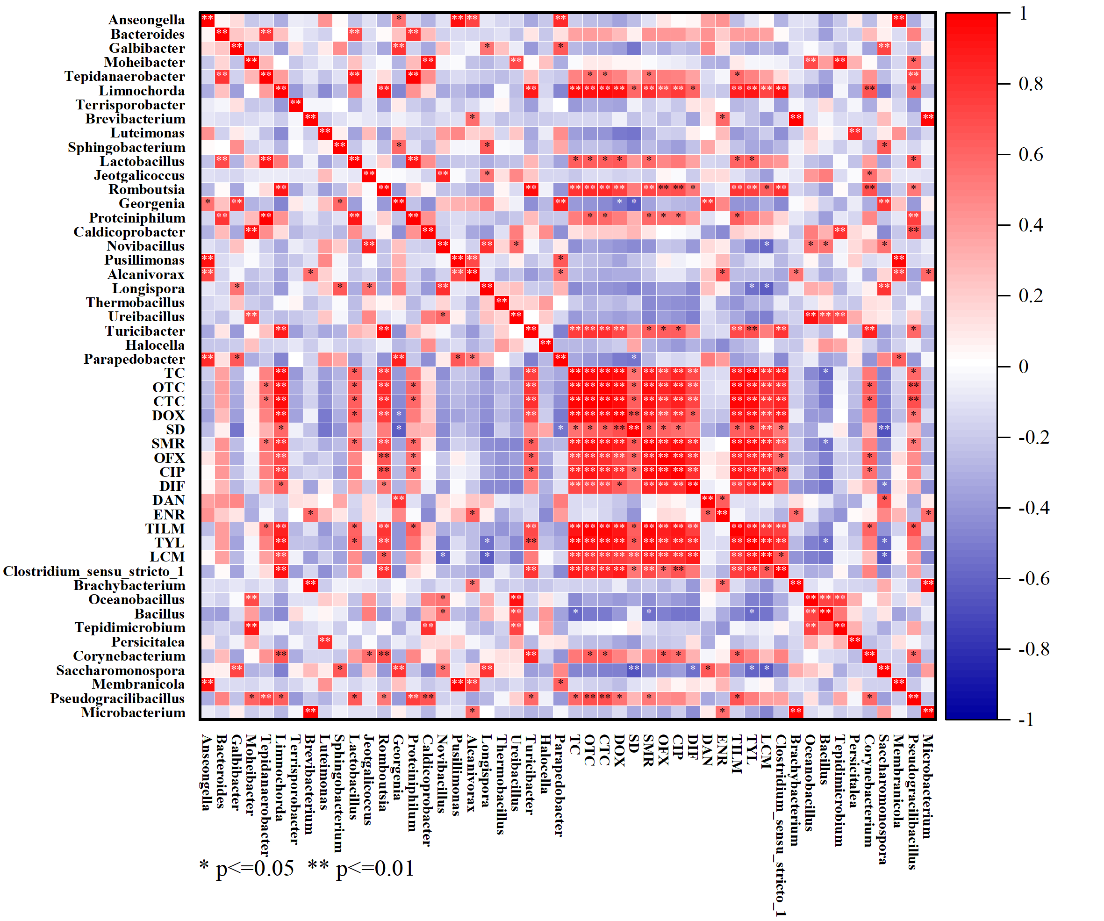


**FIGURE S4** Pearson's correlation coefficient between antibiotics and microorganisms at the genus level.

**TABLE S3** Changes in TOC, TN, C/N during composting.

| Treatment | CK | | | CK + Biochar | | |
| --- | --- | --- | --- | --- | --- | --- |
| Time (d)/Name | TOC g/kg | TN g/kg | C/N | TOC g/kg | TN g/kg | C/N |
| 1 | 423.76 ± 10.93 | 17.85 ± 0.00 | 23.74 ± 0.61 | 420.82 ± 6.99 | 16.68 ± 0.10 | 25.23 ± 0.55 |
| 3 | 368.83 ± 11.99 | 13.98 ± 0.03 | 26.38 ± 0.86 | 355.67 ± 4.62 | 16.80 ± 1.40 | 21.28 ± 1.99 |
| 6 | 338.22 ± 15.42 | 17.98 ± 0.72 | 18.85 ± 1.57 | 346.95 ± 4.37 | 17.59 ± 0.34 | 19.73 ± 0.13 |
| 10 | 331.38 ± 8.95 | 19.35 ± 0.75 | 17.14 ± 0.88 | 344.71 ± 3.42 | 16.58 ± 0.71 | 20.80 ± 0.76 |
| 14 | 314.62 ± 1.69 | 20.16 ± 0.24 | 15.61 ± 0.23 | 354.98 ± 11.54 | 17.06 ± 0.16 | 20.80 ± 0.49 |
| 21 | 298.66 ± 28.72 | 21.44 ± 2.18 | 14.02 ± 2.41 | 353.32 ± 9.37 | 16.68 ± 0.34 | 21.18 ± 0.61 |
| 28 | 278.58 ± 10.30 | 21.92 ± 1.71 | 12.75 ± 0.94 | 336.55 ± 32.91 | 19.54 ± 0.24 | 17.23 ± 1.56 |
| 35 | 293.27 ± 11.08 | 20.72 ± 0.43 | 14.15 ± 0.47 | 319.83 ± 25.18 | 19.21 ± 0.60 | 16.69 ± 1.82 |
| 42 | 300.61 ± 11.41 | 23.31 ± 1.27 | 12.91 ± 0.48 | 340.98 ± 3.70 | 18.67 ± 0.47 | 18.27 ± 0.42 |
| 50 | 262.69 ± 23.47 | 21.34 ± 1.26 | 12.35 ± 1.49 | 306.46 ± 46.77 | 22.28 ± 0.23 | 13.76 ± 2.18 |

Table S4 Absolute abundance of ARGs in different periods of composting.

| Treatment | CK | | | | | | | CK+Biochar | | | | | | |
| --- | --- | --- | --- | --- | --- | --- | --- | --- | --- | --- | --- | --- | --- | --- |
| Time (d) | 1 | 6 | 14 | 21 | 28 | 42 | 50 | 1 | 6 | 14 | 21 | 28 | 42 | 50 |
| *tetA* | 4.25E+04 | 1.82E+06 | 2.16E+07 | 4.40E+07 | 3.70E+08 | 1.94E+08 | 1.55E+08 | 2.05E+06 | 1.43E+07 | 3.95E+06 | 1.34E+08 | 2.75E+08 | 4.52E+08 | 1.81E+07 |
|  | ±1.13E+04 | ±8.31E+04 | ±1.13E+06 | ±1.27E+06 | ±1.43E+07 | ±4.49E+06 | ±1.44E+06 | ±1.15E+04 | ±4.57E+05 | ±9.35E+04 | ±1.99E+06 | ±1.92E+06 | ±1.45E+07 | ±1.91E+06 |
| *tetB* | 8.51E+05 | 1.08E+07 | 1.50E+07 | 3.21E+07 | 1.55E+08 | 1.05E+08 | 1.02E+08 | 1.48E+07 | 6.91E+07 | 0.00E+00 | 0.00E+00 | 4.29E+08 | 3.58E+08 | 0.00E+00 |
|  | ±4.84E+05 | ±1.19E+06 | ±1.01E+06 | ±3.57E+06 | ±2.74E+07 | ±1.83E+07 | ±9.47E+06 | ±2.21E+06 | ±2.00E+07 | ±0.00E+00 | ±0.00E+00 | ±1.47E+08 | ±9.33E+04 | ±0.00E+00 |
| *tetC* | 8.63E+05 | 7.73E+06 | 7.03E+06 | 5.99E+06 | 3.39E+07 | 5.28E+07 | 8.05E+06 | 9.60E+06 | 3.06E+07 | 3.63E+06 | 1.08E+07 | 2.51E+07 | 3.05E+07 | 1.36E+07 |
|  | ±6.88E+04 | ±3.27E+05 | ±4.00E+05 | ±8.16E+05 | ±7.63E+05 | ±3.17E+06 | ±2.67E+04 | ±8.03E+05 | ±2.71E+04 | ±3.74E+04 | ±1.37E+06 | ±6.96E+06 | ±6.18E+06 | ±3.82E+06 |
| *tetG* | 1.85E+05 | 3.17E+06 | 4.35E+07 | 4.41E+07 | 3.28E+08 | 2.78E+08 | 4.56E+08 | 3.75E+06 | 3.73E+07 | 1.61E+07 | 1.13E+08 | 3.57E+08 | 5.10E+08 | 4.73E+07 |
|  | ±5.96E+04 | ±9.05E+04 | ±4.27E+04 | ±4.51E+05 | ±2.16E+07 | ±4.59E+06 | ±1.50E+06 | ±6.49E+05 | ±1.68E+05 | ±3.24E+05 | ±1.05E+06 | ±1.38E+07 | ±5.26E+06 | ±1.51E+06 |
| *tetM* | 2.42E+05 | 4.98E+06 | 6.81E+06 | 1.90E+06 | 8.35E+05 | 1.47E+06 | 6.10E+05 | 1.25E+07 | 6.20E+06 | 1.18E+06 | 2.98E+06 | 2.39E+06 | 3.53E+06 | 8.99E+05 |
|  | ±3.22E+04 | ±2.37E+05 | ±6.34E+04 | ±1.20E+04 | ±1.26E+05 | ±1.14E+05 | ±1.03E+04 | ±1.66E+05 | ±3.07E+05 | ±2.80E+04 | ±5.46E+04 | ±3.22E+05 | ±1.17E+05 | ±4.40E+04 |
| *tetO* | 3.48E+05 | 1.92E+06 | 2.44E+06 | 4.61E+05 | 3.91E+05 | 4.98E+05 | 1.75E+05 | 1.26E+06 | 2.46E+06 | 1.44E+05 | 5.23E+05 | 8.73E+05 | 6.26E+05 | 6.78E+05 |
|  | ±7.04E+04 | ±8.51E+04 | ±8.34E+03 | ±1.81E+04 | ±2.55E+04 | ±2.56E+04 | ±1.63E+04 | ±3.21E+04 | ±2.72E+04 | ±2.12E+03 | ±3.44E+04 | ±1.47E+05 | ±8.79E+04 | ±1.01E+05 |
| *tetQ* | 4.41E+06 | 2.15E+07 | 4.42E+07 | 5.42E+07 | 4.21E+07 | 2.57E+07 | 1.36E+07 | 1.68E+07 | 1.23E+08 | 0.00E+00 | 0.00E+00 | 0.00E+00 | 4.17E+07 | 0.00E+00 |
|  | ±6.48E+05 | ±2.40E+06 | ±2.66E+06 | ±3.80E+06 | ±5.66E+06 | ±2.22E+05 | ±4.24E+06 | ±4.24E+06 | ±1.45E+04 | ±0.00E+00 | ±0.00E+00 | ±0.00E+00 | ±4.66E+06 | ±0.00E+00 |
| *tetW* | 1.41E+05 | 9.92E+06 | 3.33E+08 | 7.74E+06 | 7.08E+06 | 7.39E+06 | 1.68E+06 | 1.33E+06 | 1.56E+07 | 2.32E+05 | 3.85E+06 | 2.79E+06 | 7.24E+06 | 1.01E+06 |
|  | ±1.11E+04 | ±3.04E+05 | ±7.65E+06 | ±3.67E+05 | ±4.76E+04 | ±1.45E+05 | ±4.30E+03 | ±5.41E+04 | ±9.98E+05 | ±2.19E+04 | ±3.65E+05 | ±1.86E+05 | ±1.54E+04 | ±1.98E+05 |
| *tetX* | 2.17E+05 | 2.05E+06 | 1.87E+07 | 1.21E+07 | 2.60E+08 | 1.77E+08 | 7.19E+08 | 5.65E+06 | 1.75E+07 | 1.92E+07 | 2.25E+07 | 1.35E+08 | 3.57E+08 | 2.38E+06 |
|  | ±1.22E+04 | ±5.30E+04 | ±7.98E+05 | ±1.01E+06 | ±5.07E+06 | ±1.43E+06 | ±2.74E+06 | ±1.28E+04 | ±4.43E+05 | ±1.35E+06 | ±6.60E+04 | ±1.22E+06 | ±2.05E+07 | ±1.94E+05 |
| *tetZ* | 6.72E+04 | 3.68E+05 | 2.05E+06 | 1.28E+06 | 6.18E+06 | 9.02E+06 | 1.64E+07 | 2.26E+05 | 6.61E+06 | 1.05E+05 | 5.25E+06 | 1.34E+08 | 5.46E+07 | 1.13E+08 |
|  | ±4.16E+03 | ±2.93E+04 | ±8.44E+04 | ±6.63E+04 | ±7.14E+05 | ±1.27E+06 | ±8.23E+05 | ±1.08E+04 | ±6.07E+05 | ±1.08E+04 | ±5.01E+05 | ±3.37E+06 | ±8.79E+05 | ±5.48E+06 |
| *sul 1* | 3.91E+05 | 1.42E+07 | 1.40E+08 | 1.19E+08 | 8.02E+08 | 1.05E+09 | 1.48E+09 | 2.21E+07 | 1.76E+08 | 2.25E+07 | 3.56E+08 | 2.13E+09 | 2.70E+09 | 7.71E+08 |
|  | ±1.46E+05 | ±2.77E+04 | ±4.79E+06 | ±9.46E+06 | ±6.11E+07 | ±4.86E+07 | ±6.55E+07 | ±1.09E+05 | ±3.77E+06 | ±2.52E+05 | ±1.07E+07 | ±5.55E+07 | ±1.80E+07 | ±4.71E+07 |
| *sul 2* | 6.13E+05 | 1.17E+07 | 1.85E+08 | 1.23E+08 | 2.32E+09 | 2.45E+09 | 4.02E+09 | 1.24E+07 | 1.16E+08 | 3.64E+07 | 2.61E+08 | 2.80E+09 | 3.41E+09 | 6.30E+07 |
|  | ±1.23E+05 | ±6.59E+05 | ±8.41E+06 | ±2.85E+06 | ±6.33E+07 | ±7.82E+07 | ±7.71E+07 | ±1.43E+05 | ±3.13E+06 | ±2.14E+05 | ±1.33E+07 | ±4.02E+07 | ±3.71E+07 | ±5.12E+06 |
| *gyrA* | 5.26E+05 | 4.59E+06 | 3.39E+06 | 3.09E+06 | 0.00E+00 | 0.00E+00 | 0.00E+00 | 2.92E+07 | 7.63E+06 | 7.91E+06 | 0.00E+00 | 0.00E+00 | 4.93E+06 | 1.34E+07 |
|  | ±1.70E+05 | ±7.21E+05 | ±1.48E+06 | ±7.82E+05 | ±0.00E+00 | ±0.00E+00 | ±0.00E+00 | ±1.29E+07 | ±4.30E+06 | ±1.41E+05 | ±0.00E+00 | ±0.00E+00 | ±2.03E+05 | ±5.15E+06 |
| *qnrS* | 1.91E+07 | 1.06E+07 | 4.40E+07 | 8.56E+07 | 2.43E+08 | 4.37E+08 | 0.00E+00 | 7.02E+08 | 9.21E+07 | 7.16E+07 | 2.18E+08 | 3.87E+09 | 1.16E+09 | 5.47E+08 |
|  | ±1.41E+06 | ±6.85E+05 | ±5.72E+06 | ±7.07E+06 | ±2.51E+07 | ±4.24E+07 | ±0.00E+00 | ±2.69E+08 | ±4.58E+06 | ±5.66E+06 | ±4.24E+07 | ±8.44E+08 | ±5.08E+08 | ±5.66E+07 |
| *ermB* | 1.55E+06 | 1.16E+08 | 4.78E+06 | 4.75E+06 | 5.79E+06 | 0.00E+00 | 1.14E+08 | 8.19E+07 | 2.86E+07 | 3.96E+06 | 7.33E+07 | 1.62E+07 | 2.92E+07 | 7.56E+06 |
|  | ±8.91E+04 | ±8.46E+05 | ±5.51E+05 | ±1.31E+06 | ±9.95E+05 | ±0.00E+00 | ±1.26E+06 | ±8.10E+06 | ±5.52E+06 | ±3.47E+05 | ±3.47E+06 | ±2.29E+06 | ±2.78E+06 | ±1.84E+06 |
| *ermC* | 2.61E+05 | 2.81E+05 | 3.58E+05 | 7.64E+05 | 7.01E+05 | 3.40E+05 | 1.60E+06 | 3.58E+06 | 4.70E+06 | 6.57E+05 | 1.17E+08 | 2.35E+06 | 6.16E+07 | 3.71E+06 |
|  | ±3.48E+04 | ±4.03E+04 | ±1.31E+04 | ±2.99E+04 | ±1.04E+05 | ±5.61E+04 | ±2.10E+05 | ±3.57E+04 | ±1.31E+05 | ±6.56E+04 | ±5.96E+05 | ±3.33E+05 | ±1.51E+06 | ±4.44E+05 |
| *ermF* | 3.43E+05 | 2.09E+06 | 8.99E+06 | 1.65E+07 | 1.88E+08 | 2.23E+08 | 8.71E+08 | 5.98E+06 | 1.50E+07 | 1.81E+07 | 2.99E+07 | 2.20E+08 | 3.29E+08 | 1.16E+07 |
|  | ±1.77E+04 | ±6.83E+04 | ±8.77E+04 | ±4.22E+05 | ±7.62E+06 | ±4.52E+06 | ±7.59E+06 | ±1.49E+06 | ±4.71E+05 | ±6.72E+05 | ±1.67E+06 | ±7.48E+06 | ±1.59E+06 | ±3.06E+06 |
| *ermT* | 2.52E+08 | 1.69E+09 | 1.36E+09 | 1.52E+09 | 3.21E+09 | 1.49E+09 | 1.53E+10 | 2.00E+09 | 2.56E+09 | 3.26E+09 | 2.91E+09 | 6.41E+09 | 1.05E+10 | 2.73E+09 |
|  | ±1.03E+07 | ±5.92E+07 | ±9.45E+07 | ±2.99E+07 | ±1.50E+07 | ±5.56E+08 | ±2.46E+08 | ±3.20E+08 | ±5.38E+07 | ±9.60E+07 | ±1.01E+08 | ±6.38E+07 | ±7.06E+08 | ±1.34E+08 |
| *mefA* | 2.21E+05 | 4.38E+06 | 4.89E+07 | 4.59E+06 | 1.59E+06 | 4.62E+04 | 2.01E+06 | 3.06E+06 | 1.96E+07 | 4.94E+05 | 9.66E+05 | 4.49E+06 | 6.23E+06 | 1.42E+06 |
|  | ±2.67E+04 | ±1.47E+05 | ±2.38E+06 | ±3.57E+05 | ±2.26E+05 | ±0.90E+02 | ±2.97E+05 | ±1.35E+05 | ±8.18E+05 | ±4.91E+04 | ±1.38E+05 | ±6.13E+05 | ±4.19E+05 | ±4.15E+05 |
| *mphA* | 2.16E+04 | 2.07E+05 | 6.24E+05 | 6.01E+05 | 1.67E+06 | 8.38E+05 | 1.03E+07 | 1.22E+05 | 4.59E+05 | 1.66E+06 | 1.74E+06 | 1.33E+06 | 2.58E+06 | 1.13E+06 |
|  | ±3.77E+03 | ±6.70E+03 | ±6.32E+04 | ±2.02E+05 | ±5.34E+04 | ±9.49E+04 | ±4.86E+05 | ±2.71E+04 | ±1.02E+04 | ±3.76E+04 | ±3.90E+05 | ±2.01E+05 | ±1.22E+05 | ±2.05E+05 |
